# Endocervical crypt involvement by high-grade cervical intraepithelial neoplasia and its association with high-grade histopathological recurrence after cervical excision in women with negative excision margins: a systematic review and meta-analysis

**DOI:** 10.1007/s00404-023-07242-y

**Published:** 2023-10-11

**Authors:** Dimitrios Papoutsis, Martyn Underwood, William Parry-Smith, Chara Tzavara

**Affiliations:** 1 Department of Obstetrics and Gynaecology, Shrewsbury and Telford Hospital NHS Trust, Telford, UK; 2 School of Health Sciences, University of Western Macedonia, 50100 Ptolemaida, Kozani, PC Greece; 3 Department of Hygiene, Epidemiology and Medical Statistics, Medical School, National and Kapodistrian University of Athens, Athens, Greece

**Keywords:** Cervical intraepithelial neoplasia, Endocervical crypt involvement, Recurrence, Excision margins, Large loop excision of the transformation zone, Cold knife conization

## Abstract

**Background:**

There is a growing body of evidence suggesting that endocervical crypt involvement by high-grade cervical intraepithelial neoplasia (CIN) may represent a risk factor for disease recurrence after cervical treatment.

**Objectives:**

To provide a systematic review and meta-analysis on whether endocervical crypt involvement by high-grade CIN on the excised cervical specimen is associated with high-grade histopathological recurrence during the follow-up of women after cervical excisional treatment.

**Search strategy:**

We searched the Medline, Scopus, Central, and Clinical Trials.gov databases from inception till May 2023.

**Selection criteria:**

Studies that reported on women with a single cervical treatment with any method of excision for CIN2 or CIN3 lesion, negative excision margins, and whose recurrence was defined histopathologically were included.

**Data collection and analysis:**

Two reviewers independently evaluated study eligibility. We used the fixed effects model for meta-analysis.

**Main results:**

There were 4 eligible studies included in the present systematic review that evaluated 1088 women treated with either large loop excision of the transformation zone (LLETZ) or with cold knife conization (CKC). We found no significant association of endocervical crypt involvement by CIN2-3 with high-grade histopathological recurrence at follow-up after cervical excision (OR 1.93; 95% CI 0.51–3.35). The subgroup analysis of women with LLETZ cervical excision showed again no significant association with high-grade histopathological recurrence at follow-up (OR 2.00; 95% CI 0.26–3.74).

**Conclusion:**

Endocervical crypt involvement by high-grade CIN does not seem to be a risk factor for high-grade histopathological recurrence after cervical excision with negative excision margins.

**Supplementary Information:**

The online version contains supplementary material available at 10.1007/s00404-023-07242-y.

## What does this study add to the clinical work

**Table:** 

Endocervical crypt involvement has been proposed to increase the recurrence rates following cervical excisional treatment for CIN pathology. This meta-analysis showed no significant association between endocervical crypt involvement with high-grade histopathological recurrence. However, further research is needed due to the wide confidence intervals of the point estimates calculated in the meta-analysis and the potential confounders identified in the systematic review.	

## Introduction

Cervical intraepithelial neoplasia (CIN) is an abnormality of the squamous cells of the uterine cervix that may progress to cervical cancer if left untreated [1]. In accordance with the 2012 Lower Anogenital Squamous Terminology (LAST) for high-risk human papilloma virus (HPV)-related lesions of the lower genital tract, CIN2 and CIN3 lesions are grouped together as ‘high grade’ and are treated in the same way with either excisional or ablative cervical treatment [2, 3]. Current recommendations from national guidance in the United Kingdom suggest that cervical excision should aim for a depth of ≥ 7 mm from the epithelial surface since the endocervical crypts (or glands) extend to a maximum depth of 5.22 mm from the surface of the cervix [4–6]. In the case of cervical ablation, there is literature evidence showing that the depth of tissue destruction is 4–7 mm, thus providing equivalent cure rates when compared to cervical excision [7, 8]. However, despite cervical treatment, approximately 15% (range: 5–25%) of women will develop high-grade recurrence with 80% of these cases occurring within the first 2 years of follow-up [9–11].

There are many risk factors that have been suggested in the literature as predictive of disease recurrence such as incomplete excision margins, age, depth of excision, CIN grade, and persistent HPV infection [12–16]. Since 1990, there are reports that endocervical crypt involvement by CIN may represent a new independent risk factor for CIN recurrence [17–19, 19]. Endocervical crypt involvement has been reported to be associated with a twofold to threefold increased risk of CIN recurrence in several studies [20–23], and in the case of expansile crypt involvement defined as extensive involvement and expansion of the underlying crypts with a fourfold increased risk of recurrence [24]. Some studies, however, report that endocervical crypt involvement is not associated with subsequent recurrence [15].

The present systematic review and meta-analysis aims to assess whether endocervical crypt involvement by high-grade CIN on the excised cervical specimen is associated or not with histopathologically confirmed CIN2-3 recurrence at follow-up in women who were treated with cervical excision and with negative excision margins.

## Methods

This systematic review and meta-analysis was designed according to the Preferred Reporting Items for Systematic Reviews and Meta-Analyses (PRISMA) guidance. Τhe protocol has been registered with the PROSPERO register (CRD42021258989).

### Eligibility criteria

The eligibility criteria for the inclusion of studies were predetermined. We included all observational studies that assessed the high-grade histopathological recurrence at follow-up in women who had a single excisional cervical treatment and their excised cervical tissue specimen showed endocervical crypt involvement by CIN2 or CIN3 and negative excision margins. Studies that reported no histopathological data of recurrence after excision, or included women with a previous ablative or excisional treatment were excluded.

### Search strategy

The literature search was conducted using the Medline, Scopus, Cochrane Central Register of Controlled Trials (CENTRAL), and Clinical Trials.gov databases, together with the reference lists of electronically retrieved full-text papers. No date or language restrictions were applied. The search was performed from inception, and the date of the last search was May 31, 2023. The literature search was based on the following terms: "Endocervical crypt involvement", "Endocervical gland involvement", "Endocervical glandular involvement", "Large loop excision of the transformation zone (LLETZ)", "Cold knife conization (CKC)", "Excision margins", "Recurrence", "Histological recurrence", and "Histopathological recurrence".

### Study selection

The studies were selected in the following consecutive stages. First, the titles and abstracts of all electronic articles were screened by two authors (DP and CT) to assess their eligibility. Second, the decision for including studies in the present systematic review and meta-analysis was taken after retrieving and reviewing the full text of articles that were considered eligible. Third, any potential discrepancies in the stage of retrieval of studies and statistical analyses were resolved by the consensus of all authors.

### Data collection

The following data were extracted from each of the included studies: name of the first author, year of publication, country, study design, sample size, eligibility criteria, demographic data, cervical treatment features, follow-up data, and the primary outcome of interest. The data extraction was independently conducted by two researchers (DP and CT), while any outcomes or disagreements were resolved by their consensus or by discussion with all authors.

### Definitions

We included in the meta-analysis studies that defined high-grade histopathological recurrence as the histopathological finding of CIN2 or CIN3 on cervical punch biopsy or repeat cervical excision or subsequent hysterectomy during the follow-up of women who underwent an initial cervical excision.

### Outcome measures

The primary outcome was to identify whether the presence or absence of endocervical crypt involvement by CIN2 or CIN3 on the excised cervical specimen is associated with high-grade histopathological recurrence at follow-up after cervical excision in women with negative excision margins. For this reason, women from all included studies in the meta-analysis and with endocervical crypt involvement by CIN2-3 on the excised cervical specimen were compared against women without endocervical crypt involvement by CIN2-3 on the excised cervical specimen. A subgroup analysis was conducted for women who underwent LLETZ cervical excision.

### Data analysis

Fixed effects meta-analysis was conducted for the primary outcome to derive pooled odds ratios (OR) with 95% confidence intervals (CI). In the case that no events were observed in one arm of an individual study that was included in the meta-analysis, the calculation of the odds ratios was based on continuity correction [25]. The presence of heterogeneity was assessed by means of a test on the Q statistic and we calculated the I^2^ index. If I^2^ values were more than 50%, we considered the data significantly heterogeneous [26]. Statistical significance was considered at *p* < 0.05. All statistical analyses were performed with STATA software version 14.0.

### Risk of bias within studies assessment

Risk of bias within studies assessment included review of seven domains based on the ROBINS-I risk of bias tool [27]. The domains included bias due to confounding, bias in selection of participants into the study, bias in classification of the intervention, bias due to deviations from intended interventions, bias due to missing data, bias in measurement of outcomes, and bias in selection of the reported result. Risk of bias assessment was completed independently by two researchers (DP, CT). Disagreements were resolved by consensus meeting.

### Certainty of evidence assessment

We assessed the level of certainty of the evidence using the GRADE approach [28]. Depending on assessments for risk of bias, indirectness of evidence, serious inconsistency, imprecision of estimates and potential publication bias, the level of certainty for the evidence provided can be downgraded from 'high' by one level to ‘low’ for serious concerns, or by two levels to ‘very low’ for very serious concerns.

## Results

### Study selection

**Fig. 1:**
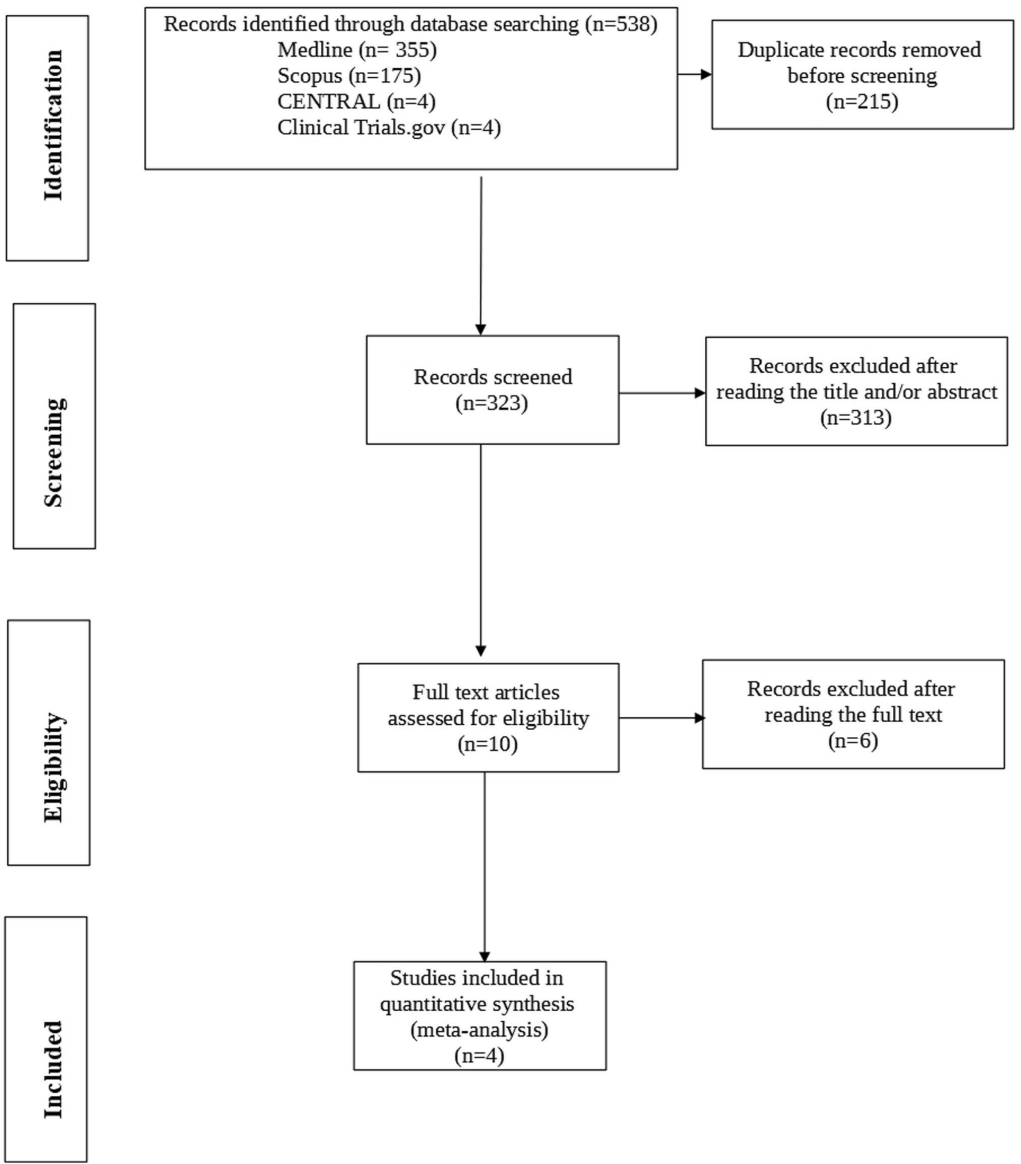
The PRISMA flow chart of study selection

The process of study selection is schematically depicted in the PRISMA flowchart (Fig. 1). Overall, a total of 538 papers were retrieved from Medline (*n* = 355), Scopus (*n* = 175), Cochrane Central Register of Controlled Trials (CENTRAL) (*n* = 4), and Clinical Trials.gov databases (*n* = 4). After removing 215 duplicates, 323 publications were screened for title and abstract. After reading the title and/or abstract, 313 were excluded and 10 articles were assessed for eligibility. Three authors were contacted for further information and only one responded. Finally, four eligible articles reporting four studies were included in the systematic review [18, 21–23].

### Included studies

The methodological characteristics of the four eligible studies included in the current systematic review are presented in Tables 1 and 2. All retrieved studies were observational studies and cervical treatment was conducted with either LLETZ or CKC excision. Endocervical crypt involvement by CIN2 or CIN3 on the cervical excision specimen with negative margins ranged between 28.4% and 50.7%. The CIN2-3 recurrence rate defined histopathologically at follow-up after excision in these four studies ranged between 0.5% and 4.8%, with a minimum follow-up of 2 years after treatment (range: 2–9 years).

**Table 1:** Study characteristics

Study	Continent of origin	Type of study	Study sample	Women with negative margins and CIN2 + on cone specimen (no invasive cancer)	Mean age of women in study	Follow-up after cervical excision	Definition of recurrence
Demopoulos et al. (1991) [18]	USA	Observational	*n* = 341	*n* = 204	34 yo	Treatment: 1979–1983 Followed up till 1988	Histopathological
Kodampur et al. (2013) [23]	UK	Observational	*n* = 309	*n* = 309	30 yo	Treatment: 2003–2004 Followed up till 2010	Histopathological
Papoutsis et al. (2015) [22]	UK	Observational	*n* = 526	*n* = 187	36.6 yo	Treatment: 2010–2011 Followed up till 2013	Cytological and Histopathological
Spinillo et al (2020) [24]	Italy	Observational	*n* = 1301	*n* = 388	39 yo (with ECI) vs 38 yo (without ECI)	Time period of study: 2010–2018	Histopathological

*ECI* endocervical crypt involvement

**Table 2:** Cervical treatment features

Study	Method of excision	Size of cervical excision in total sample	Women with negative margins and CIN2 + on cone specimen (no invasive cancer)	CIN grade on excised tissue specimen	Percentage of endocervical crypt involvement (%) (with negative margins)	CIN2 + histopathological recurrence rate	CIN2 + histopathological recurrence at follow-up after excision (with ECI vs without ECI) (No Ca at follow-up)
Demopoulos et al. (1991) [18]	CKC	2.2/1.5 cm (mean diameter/height)	*n* = 204	CIN3	58/204 (28.4%)	9/204 (4.4%)	2/58 (3.4%) vs 7/146 (4.8%)
Kodampur et al. (2013) [23]	LLETZ	N/A	*n* = 309	CIN2, CIN3	117/309 (37.9%)	11/309 (3.5%)	6/117 (5.1%) vs 5/192 (2.6%)
Papoutsis et al. (2015) [22]	LLETZ	1.2 cm (median cone depth)	*n* = 187	CIN2, CIN3	61/187 (32.6%)	1/187 (0.5%)	0/61 (0%) vs 1/126 (0.7%)
Spinillo et al (2020) [24]	LLETZ and CKC	N/A	*n* = 388	CIN2, CIN3	197/388 (50.7%)	19/388 (4.8%)	19/212 (8.9%) vs 7/176 (3.9%)

*ECI* endocervical crypt involvement, *LLETZ* large loop excision of the transformation zone, *CKC* cold knife conisation, *N/A* not applicable

### Risk of bias within studies assessment (ROBINS-I tool)

Based on the ROBINS-I risk of bias assessment tool, three of the studies included in the review were of moderate risk of bias and one study was of low risk of bias (Table S1).

### Certainty of evidence assessment (GRADE)

When using the GRADE criteria for assessing whether crypt involvement on the cervical excision specimen is a predictor of recurrence at follow-up, the certainty of the evidence begins at the level of “low” because all the research included in the analyses is conducted as observational studies. We found that the level of certainty of evidence in our review was ‘low’ and was further downgraded to ‘very low’ due to the wide confidence intervals of the precision estimate that was calculated in the meta-analysis (Table S2).

### Quantitative synthesis

**Fig. 2:**
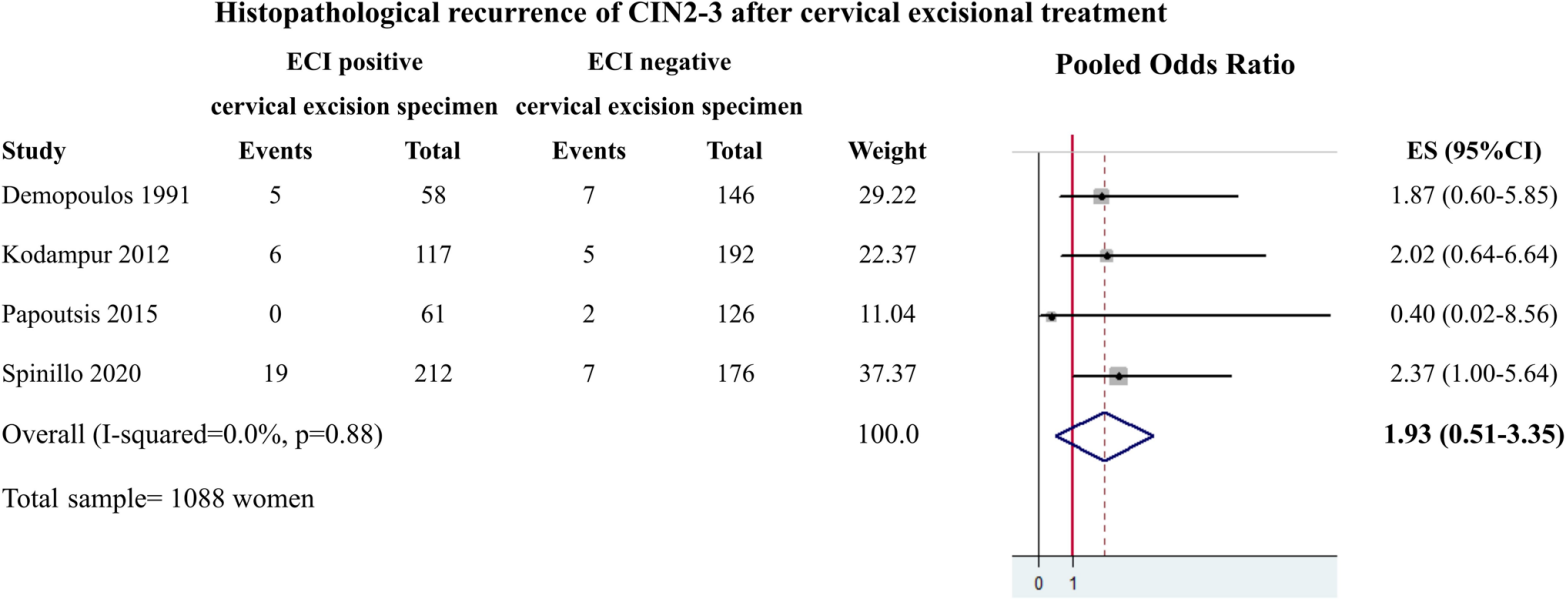
Results from the fixed effects meta-analysis including all 4 studies (*N* = 1088 women)

**Fig. 3:**
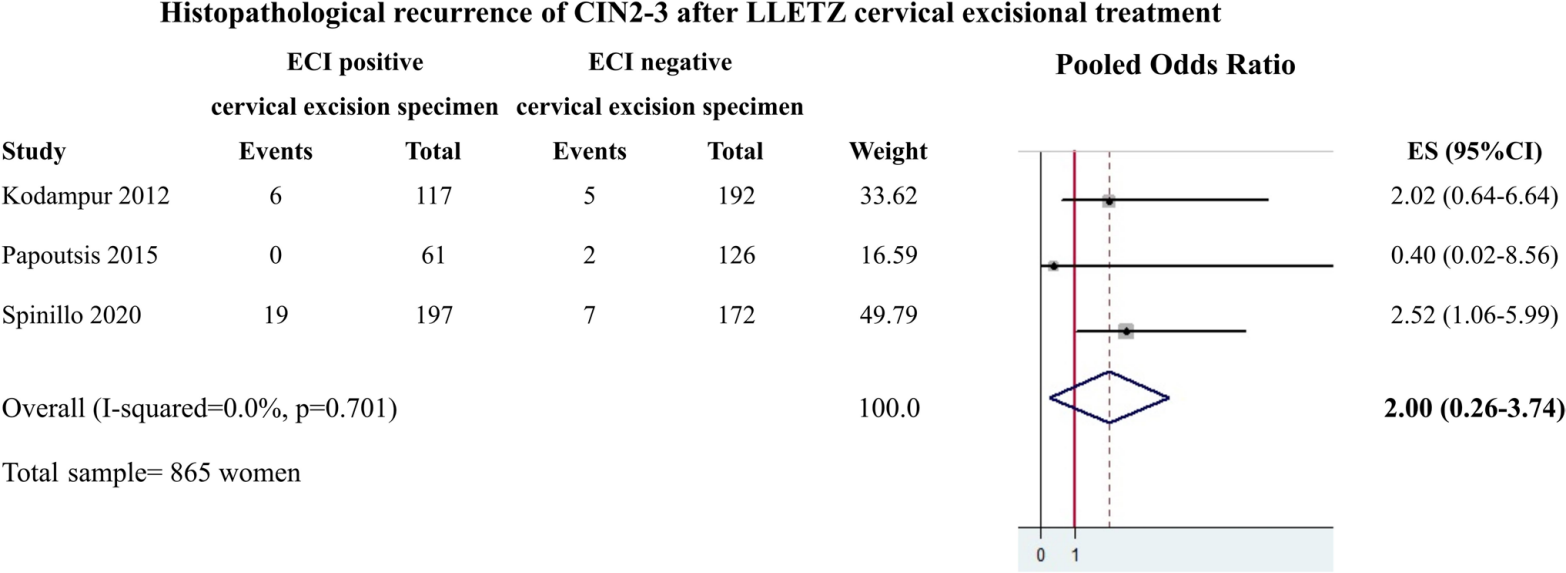
Results from the fixed effects meta-analysis including 3 studies with LLETZ cervical excision (*N* = 865 women)

The meta-analysis was based on the results from the 4 included studies and involved a total of 1088 participants. The between-study heterogeneity was not significant (*Q* = 0.64, *p* = 0.888, *I*^2^ = 0.0). Therefore, a fixed effects model was used to calculate the pooled OR that was found to be 1.93 (95% CI 0.51–3.35), indicating no significant association of endocervical crypt involvement by CIN2-3 with high-grade histopathological recurrence (Fig. 2). A subgroup analysis of cases with LLETZ as method of excision showed a pooled OR equal to 2.00 (95% CI 0.26–3.74), indicating no significant association of endocervical crypt involvement by CIN2-3 with high-grade histopathological recurrence (Fig. 3).

## Discussion

### Main findings

The present systematic review showed that there is no significant association of endocervical crypt involvement by CIN2-3 on the excised cervical tissue specimen with CIN2-3 histopathological recurrence in women treated with cervical excision and negative excision margins (OR 1.93; 95% CI 0.51–3.35). This finding remained even after a subgroup analysis was conducted for women treated with only LLETZ cervical excision. The level of certainty of evidence for these findings is considered as ‘very low’ due to the observational nature of the included studies and the wide confidence intervals of the precision estimate that was calculated in the meta-analysis.

### Strengths and limitations

Based on our literature search, this is the first systematic review and meta-analysis on this topic. However, there are certain limitations to be considered about the systematic review and meta-analysis. First, the use of strict inclusion criteria in our systematic review resulted in the limited number of studies being included. Second, in our meta-analysis, it was not possible to perform subgroup analyses based on the excised cervical depth as this information was not available for half the included studies. Moreover, all the cervical excision studies in the literature that explore the association of endocervical crypt involvement with CIN recurrence describe the overall crypt involvement and do not report on the existence of expansile crypt involvement. There is only one study in the literature that involved cold coagulation ablative treatment suggesting that it is not the non-expansile crypt involvement by CIN2-3 element but the expansile crypt involvement by CIN2-3 on cervical tissue that increases by fourfold the recurrence rate [24]. Finally, the NHS-CSP guidance on how to report histopathology results has been in place since 2012 in the United Kingdom necessitating the documentation of the presence of crypt involvement in all histopathology reports and to use the term expansile involvement when the histopathologic criteria are met [24, 29]. It could be that if expansile crypt involvement had been reported in all the cervical excision studies, then the results of the meta-analysis may have been different.

### Interpretation

There are reports as early as 1976 suggesting that recurrence of CIN in women treated with cervical excision and with negative excision margins may be the result of involvement of the glands deep in the endocervical canal [30]. Other researchers in the decades of 1980, 1990, and 2020’s speculated that the remaining of islands of dysplastic epithelium following cervical treatment that are concealed under the surface epithelium for some time may explain the recurrence of disease [18, 31, 32]. The question that has been raised is whether the recurrence of CIN results from de novo malignant transformation of the regenerated epithelium after cervical treatment or if it originates from the microscopic failure to remove all CIN disease even when excision margins are reported as clear.

The literature states that when CIN pathology is detected within 2 years of treatment, then it is believed to be a treatment failure and CIN was not removed completely at primary treatment [33]. When CIN is discovered after 3 years of treatment, then it is considered to be a re-occurrence [34]. An observational cohort study in 2013 investigated the early and late recurrence of women who had LLETZ treatment for CIN2-3 and the cervical tissue specimen showed negative excision margins [22]. In that study, women who were crypt-positive had a gradual and early rise in recurrence within 2 years from treatment, whereas the crypt-negative group had a late and sharp rise in recurrence after 2 years from treatment. The authors concluded that this pattern of recurrence suggests that residual CIN in crypts in ‘presumed completely excised CIN’ may play a role in the early disease recurrence, whereas the late disease recurrence is most likely attributable to new disease occurrence than a comeback of CIN hidden in crypts [22].

There are reports that HPV persistence at 6 months following cervical excision is an independent risk factor that increases by 20-fold the likelihood for CIN recurrence [15]. In a recent study with HPV testing of the participants, it was found that endocervical crypt involvement-positive women when compared to endocervical crypt involvement-negative women were significantly associated with the presence of HPV 16 infection (38.1% vs 26.7%; *p* < 0.05) and multiple high-risk HPV infection (42.9% vs 32%; *p* < 0.05) at follow-up after cervical excision [23]. It is well documented that HPV 16 infection is the main driver of cervical oncogenesis while multiple high-risk HPV infection is associated with increased extension and severity of CIN lesions as well as increased rates of recurrence of CIN after treatment [23, 34–36].

With regard to the depth of a CIN lesion involving the endocervical crypts, a study found that histopathologically the mean depth of CIN2 gland involvement was 0.8 mm (range 0.2–5 mm) while that of CIN3 was deeper at 1 mm (range 0.2–7.5 mm) (*p* = 0.039) [15]. Moreover, in women with CIN2-3 on pretreatment cervical punch biopsy and endocervical crypt involvement on excised tissue, the high-grade cytology recurrence was significantly reduced if more than 1.9 cm^3^ of cervix was removed [21]. It, therefore, seems plausible that when there is endocervical crypt involvement, the depth of cervical tissue removed should be relatively greater compared to cervical tissue with no crypt involvement. Nevertheless, it should be considered that this removal of relatively more cervical tissue with crypt involvement so as to ensure lower oncological recurrence rates needs to be balanced against the risk of higher spontaneous preterm birth rates that have been reported in the future pregnancies of these women [1, 37].

Our meta-analysis has found no effect of endocervical crypt involvement on the high-grade recurrence of CIN after excision, despite the reports in the literature that crypt involvement increases the recurrence rates after treatment and the suggested hypothesis that this could represent a deeper CIN lesion with a more aggressive potential due to the presence of high-risk HPV strains. The explanation we can provide for our findings is that the excision depth in the studies included in the meta-analysis was adequate so as to remove these presumed deeper lesions that are associated with high-risk HPV strains. Unfortunately the excision depth cannot be accounted for across the included studies nor can the extent of the lesion size on the ectocervix be determined so as to include in the meta-analysis.

The studies included in the meta-analysis reported on CIN2 and CIN3 on the excised tissue specimen and on the recurrence post-treatment. However, even though CIN2 and CIN3 are considered both as “high-grade”, they nevertheless demonstrate different rates of progression to cervical cancer and possess varying rates of spontaneous regression. For biopsy proven but untreated CIN3, the risk of progression to cervical cancer has been reported to be about 40% (1% annually) in England and Wales and 15–23% in Sweden [38, 39]. On the other hand, there is evidence that CIN2 lesions have a high rate of spontaneous regression and should not always be managed with an immediate intervention as in the case of CIN3 but could be managed conservatively. For women under 25 years of age with biopsy-proven CIN2, the spontaneous regression rates described in the literature range between 59 and 68% [40, 41], with annual regression rates being reported of 15–23% [42]. For women over 25 years of age with biopsy-proven CIN2, the spontaneous regression rates described vary between 40 and 74% [43, 44]. Since the studies included in our meta-analysis involved cases with both CIN2 and CIN3, the finding of no significant effect of crypt involvement on the recurrence of CIN at follow-up could potentially reflect this spontaneous regression potential of CIN2. It has been documented that expansile endocervical crypt involvement by high-grade CIN was associated with CIN2 in one-third of women and with CIN3 in two-thirds of women [24]. Unfortunately there was no clear information across the included studies so as to separate cases with CIN2 from CIN3 in the analyses.

## Conclusion

Our meta-analysis has shown that endocervical crypt involvement by CIN2-3 on the excised tissue specimen with negative excision margins is not significantly associated with CIN2-3 recurrence at follow-up. However, further research is required in the field of cervical excision with the inclusion of women with CIN3 only due to the high spontaneous regression rates of CIN2 on cervical tissue, and with the histopathological identification of the expansile crypt element that has been shown in cervical ablation studies to increase by fourfold the recurrence rate.

### Supplementary Information

Below is the link to the electronic supplementary material.

Supplementary file1 (DOC 25 KB)

Supplementary file2 (DOC 26 KB)

## Data Availability

Data sharing not applicable to this article as no datasets were generated or analyzed during the current study.
