# Supplementary material for: Endocervical crypt involvement by high-grade cervical intraepithelial neoplasia and its association with high-grade histopathological recurrence after cervical excision in women with negative excision margins: a systematic review and meta-analysis
Source: Arch Gynecol Obstet. 2023 Oct 11;309(3):939–48. doi: 10.1007/s00404-023-07242-y (PMC10867046; doi:10.1007/s00404-023-07242-y)
Supplement: Supplementary file 1 — Supplementary file1 (DOC 25 KB) [file 404_2023_7242_MOESM1_ESM.doc]

**Table S1.** ROBINS-I assessment tool

| Study | Bias due to confounding | Bias in selection of participants into the study | Bias in classification of intervention | Bias due to deviations from intended interventions | Bias due to missing data | Bias in measurement of outcomes | Bias in selection of reported result | Overall bias |
| --- | --- | --- | --- | --- | --- | --- | --- | --- |
| Demopoulos et al (1991)^18^ | Moderate | Moderate | Low | Low | Low | Low | Low | Moderate |
| Kodampur et al (2013)^23^ | Low | Low | Low | Low | Low | Low | Low | Low |
| Papoutsis et al (2015)^22^ | Moderate | Low | Low | Low | Low | Moderate | Low | Moderate |
| Spinillo et al (2020)^24^ | Moderate | Low | Low | Low | Low | Low | Low | Moderate |

****Interpretation:****

The ROBINS-I tool covers seven domains through which bias might be introduced. The first two domains address issues before the start of the interventions that are to be compared (“baseline”) and the third domain addresses classification of the interventions themselves. The other four domains address issues after the start of interventions.

In our review, the term ‘intervention’ has the meaning of the term ‘exposure’, which essentially refers to the presence or not of crypt involvement in the women who are followed up to determine their outcome (recurrence) in each study included in the review.

1) Bias due to confounding: Baseline confounding occurs when one or more variables (factors) that predict the outcome of interest (CIN recurrence at follow-up) also predict the exposure at baseline (presence or not of crypt involvement). Since a confounding effect was expected because of the factor of excision margin status in the cervical tissue specimen, the women included in the meta-analysis all had negative excision margins. The study of Kodampur et al included only women with negative excision margins, whereas the other three studies in the review included all women irrespective of margin status but did provide information for negative excision margin status that was used in the meta-analysis.

2) Bias in selection of participants into the study: When exclusion of some eligible participants is related to both the exposure (crypt involvement) and the outcome (recurrence), there will be an association between exposure and outcome even if the effects of the exposure or no-exposure are identical. In this case, the study of Demopoulos et al excluded women with CIN2 lesions and included only women with CIN3 lesions. The other three studies included women with both CIN2 and CIN3 on the cervical excision specimen.

3) Bias in classification of intervention: In all four included studies, the exposure which related to the presence or not of crypt involvement was clearly defined.

4) Bias due to deviations from intended interventions: This bias arises when there are systematic differences between exposure and non-exposure groups in the care provided to them. In the systematic review, all four included studies reported that all women after their cervical excision were followed-up for possible recurrence.

5) Bias due to missing data: This bias arises when follow-up is missing for individuals initially included and followed. In all four studies, the loss to follow-up rates of women were considered low.

6) Bias in measurement of outcomes: This bias is introduced by potential errors in measurement of outcome data. Such bias can arise if different methods are used to assess outcomes in different participant groups. In this case, the study of Papoutsis et al in their original article measured cytology recurrence of women with and without crypt involvement and also provided histopathological data of women at follow-up. For the purposes of the review, additional analyses were requested and conducted by Papoutsis et al to provide data with regards to histopathological recurrence and crypt involvement. The other three studies included women who were followed up for histopathological recurrence.

7) Bias in selection of reported result: This bias is introduced when there is selective reporting of results in a way that depends on the findings, which did not apply for the included studies in the review.

**Conclusion**:

Overall bias: Based on the ROBINS-I risk of bias assessment tool, three studies were of moderate risk of bias and one study was of low-risk of bias.
