# Supplementary material for: Endocervical crypt involvement by high-grade cervical intraepithelial neoplasia and its association with high-grade histopathological recurrence after cervical excision in women with negative excision margins: a systematic review and meta-analysis
Source: Arch Gynecol Obstet. 2023 Oct 11;309(3):939–48. doi: 10.1007/s00404-023-07242-y (PMC10867046; doi:10.1007/s00404-023-07242-y)
Supplement: Supplementary file 2 — Supplementary file2 (DOC 26 KB) [file 404_2023_7242_MOESM2_ESM.doc]

**Table S2.** GRADE (certainty of evidence) approach

|  | | | **Reduce Grade** | | | | | **Increase Grade** | | |  |
| --- | --- | --- | --- | --- | --- | --- | --- | --- | --- | --- | --- |
| **Outcome** | **Studies** | **Sample size** | **Risk of Bias** | **Inconsistency** | **Indirectness** | **Imprecision** | **Publication Bias** | **Magnitude** | **Dose-Effect** | **Plausible Confounding** | **Final Grade** |
| Recurrence  (all methods) | 4 | 1088 | - | - | - | **↓** | - | - | - | - | ⊕ΟΟΟ  Very Low |
| Recurrence (LLETZ only) | 3 | 865 | - | - | - | **↓** | - | - | - | - | ⊕ΟΟΟ  Very Low |
| **GRADE Working Group grades of evidence**  **High certainty**: we are very confident that the true effect lies close to that of the estimate of the effect.  **Moderate certainty**: we are moderately confident in the effect estimate. The true effect is likely to be close to the estimate of the effect, but there is a possibility that it is substantially different.  **Low certainty**: our confidence in the effect estimate is limited. The true effect may be substantially different from the estimate of the effect.  **Very low certainty**: we have very little confidence in the effect estimate. The true effect is likely to be substantially different from the estimate of effect. | | | | | | | | | | | |

**Interpretation:**

When using the GRADE Criteria for assessing whether crypt involvement on the cervical excision specimen is a predictor of recurrence at follow-up, the certainty of the evidence begins at the level of “low” because all the research included in the analyses is conducted as observational studies.

**Reduce Grade:**

**1) Risk of bias** is low due to the strict criteria used for inclusion of studies. A potential source of bias may potentially arise from the loss of women at follow-up which in the included studies is however considered to be low and therefore does not downgrade the certainty of the findings.

**2) Inconsistency of estimates between studies** is not present as they have a similar design since they all use excisional methods of cervical treatment, and they have all documented the presence or not of crypt involvement on the cervical excision specimen with negative margins. Statistical heterogeneity is also low as measured with the I^2^ statistic.

**3) Indirectness of the evidence** reduces certainty when the population studied is not the population for the intended review and thus the applicability of findings is affected. In this review, all studies included women having had cervical excision and high-grade CIN with negative excision margins on the cervical specimen, and who were followed up for recurrence. The criterion of indirectness therefore does not affect the certainty of our evidence.

**4) Imprecision of the estimate** for a systematic review is generally measuring the ability of the evidence to find a statistically significant result. The review included a sample of 1088 total events and found that the OR for risk of recurrence in women with crypt involvement versus women without crypt involvement was OR=1.93 (95%CI:0.51-3.35). This finding was not statistically significant and showed a wide confidence interval, hence downgrading the certainty of the evidence from “low” to “very low”.

**5) Publication bias** for outcomes of the included studies was visually assessed by a funnel plot, and the asymmetry of the funnel plots was further assessed using the Egger statistical test. The results showed no significant publication bias.

**Increase Grade:**

1) Certainty of evidence is upgraded when the **magnitude of effect** is large and consistent among the studies. Even though we found an effect size of OR=1.93, however the confidence interval was wide and the result was non-significant.

2) Certainty of evidence is upgraded when the evidence suggests a **dose-effect**. This does not apply to our review.

3) Certainty of evidence is upgraded when controlling for potential sources of **confounding** are likely to result in a more favorable outcome. In the review and as explained in the limitations, we could not control across the included studies for factors such as lesion size, depth of cervical excision, and the presence of expansile crypt involvement. If we had been able to control for all these confounding factors, then the certainty of evidence irrespective of the effect size and direction would have been increased. Further research is warranted to clarify this.

**Conclusion**:

We found ‘low’ certainty evidence due to the observational nature of the included studies, which was further downgraded to ‘very low’ certainty evidence due to the wide confidence intervals of the precision estimate. This imprecision warrants additional research to determine the effects of crypt involvement on the recurrence of women at follow-up after cervical excision.
